# Supplementary material for: Risk of recurrent venous thromboembolism in patients with HIV infection: A nationwide cohort study
Source: PLoS Med. 2020 May 14;17(5):e1003101. doi: 10.1371/journal.pmed.1003101 (PMC7224453; doi:10.1371/journal.pmed.1003101)
Supplement: S1 Protocol — (DOCX) [file pmed.1003101.s002.docx]

**Template research proposal - etiology**

Proposal number: A050

Title/subject: HIV-infection as a risk factor for recurrent VTE

Key words: HIV, venous thrombosis, recurrence

Date: 11-10-2016

Researcher(s):

UMC Groningen: Vladimir Tichelaar, W. Bierman, J. Borjas-Howard.

Erasmus MC Rotterdam: B. Rijnders, C. Rokx.

Leiden UMC: Willem Lijfering, Suzanne Cannegieter

1. **Background and relevance**
   1. *Hypothesis*

Patients with an active HIV-infection have a 2-6 fold increased risk of venous thromboembolism (VTE) as compared with the general population [1-4]. Currently, very few data exist if HIV-infected patients are at increased risk for recurrent VTE. A pilot study reviewing available literature yielded only 4 case reports on recurrent VTE in HIV-infected patients [5-8]. However, with evidence of a hypercoagulable state in HIV-infected patients that is only partially restored with anti-retroviral therapy [9-11], we hypothesize that incidence of VTE recurrence is increased in HIV-infected patients.

- 1. *Objectives*
- To assess the incidence of recurrent VTE in a cohort of patients with an active HIV-infection and a history of HIV-associated first VTE.
- To determine the increase in risk of recurrent VTE in active HIV-infected patients as compared with consecutive patients with VTE enrolled from Dutch anticoagulation clinics.
- To explore whether certain HIV-specific markers of viremia/immune activity are associated with an increased risk of recurrent VTE. These risk factors are:
  - HIV-RNA load: detectable versus undetectable
  - CD4/CD8 + T-cell counts

1. **Study design**
   1. *Population description (inclusion/exclusion criteria)*
      1. *Cohort of patients with HIV*

In the Netherlands, the SHM (Stichting HIV Monitoring) follows all consenting HIV-infected patients from diagnosis until death or migration. Every time a patient visits his/her HIV outpatient clinic data are collected and registered. These include: (changes in) medication, medical history, hospital admissions, co-infections, co-morbidity (in particular risk factors for cardiovascular disease), and any complications and side effects of anti-retroviral therapy. Additionally, adverse events by CDC classification such as cancer diagnosis, hospitalization and surgery are collected. We collected data from 14,386 HIV-1 infected patients from January 2003 through March 2015 by analysis of the SHM database. 229 of these had an objectively identified first VTE according to our initial adjudication, which considers VTE of the deep legs veins (popliteal or more proximal), pulmonary embolism, arm veins (subclavian vein or more proximal), splanchnic veins or cerebral veins. For analysis of recurrent VTE, we only considered DVT of the legs and pulmonary embolism as index event.

- - 1. *Cohort from Dutch anticoagulation clinics**

Consecutive patients aged 18 to 70 years with a first proximal DVT and/or PE were included in the Multiple Environmental and Genetic Assessment of risk factors for venous thrombosis (MEGA) study. Details of the MEGA study have been described previously [12, 13]. In short, 4956 patients were recruited in the period between February 1999 to September 2004 by 6 Dutch anticoagulation clinics where at the time all patients with VTE who received treatment with vitamin K antagonists were registered.

Of the patients included, 225 did not consent to participate in a follow-up study on recurrent venous thrombosis. Thus, 4731 patients were followed from their first venous thrombotic event until 2008-2010 when they completed a questionnaire on recurrent venous thrombotic events [14]. Furthermore, between 2007 and 2009 the vital status of all patients was acquired from the central Dutch population register [15]. For the patients who died, the cause of death (ICD-10-CM encoded) was obtained from the national register of death certificates at the Central Bureau of Statistics. Deaths due to recurrent venous thrombosis were counted as fatal recurrent events. Information from the anticoagulation clinics, hospital discharge letters, questionnaires filled in by the patients and death certificates was combined and based on this, recurrences were classified into certain and uncertain recurrences, following a decision rule as published previously [14].

- 1. *Exposure (independent variable)*

In the current study, the exposure is HIV-infection. This is defined as having the diagnosis of HIV-1/2 infection (i.e. for the SHM cohort: being registered in the SHM database) with or without the use of anti-retroviral therapy. Participants in the MEGA cohort that are known to have HIV-1 or HIV-2 infection or that have been using any type of antiretroviral drugs as determined by linkage with SFK, will be excluded from the current analysis for this study.

- 1. *Outcome (dependent variable)*

The outcome of the current study is recurrent venous thrombosis (VTE). We will use the definition of this outcome from the MEGA cohort and adjust adjudication of recurrent events in the SHM cohort accordingly.

- - 1. *Outcomes and definition of outcomes in the HIV-cohort*

Due to the fact that VTE was not a systematically adjudicated outcome event in the SHM cohort, index VTE cases were identified in the SHM cohort by systematically reviewing charts of patients who had recorded use of anticoagulation: coumarines (fenprocoumon or acenocoumarol), low molecular weight heparin, unfractioned heparin, direct thrombin inhibitors (dabigatran) or factor Xa inhibitors (rivaroxiban, apixaban) or, when thrombolysis (alteplase, urokinase, ATC codes: 249, 267, 758, 801, 821, 855) had been administered PE with obstructive shock was considered. VTE was adjudicated when objective imaging report was available demonstrating DVT (in the deep veins starting at the popliteal vein or above) or PE. When imaging was not available, VTE was adjudicated if

1. the charts clearly indicated VTE as the reason for anticoagulant use
2. there were no clear indications that the anatomical location of VTE was in superficial veins, calf veins or that the diagnosis was made only on clinical grounds.
3. Anticoagulation use was documented for at least 3 months

The strategy was identical for recurrent VTE. However, VTEs for which recurrence diagnosis might be questionable (suspected recurrent DVT in the same leg as the index DVT, pulmonary embolism not clearly demonstrated in a different vascular site than the first PE), were adjudicated through independent subjective assessment by 3 authors (WB, YT, CR), to classify VTE as unlikely, probable or definite. All available diagnostic clinical details (laboratory assessment, imaging report) were made available to the authors. The assessors were blinded to the therapeutic decision made in these individual cases, as this may bias their adjudication assessment. See appendix 1 for the full definitions of outcomes and the adjudication strategy.

The observation period for a recurrent VTE will start at the withdrawal date of anticoagulant treatment for the first VTE and last until one of the following events occurs: the outcome of interest, death or last follow up date. For 8% of the index events in the SHM cohort, the margin of error for withdrawal date was greater than 3 months. For these cases, we assigned a date in the middle of the earliest and the latest possible date of withdrawal of anticoagulant therapy. Sensitivity analyses in which follow up time using the earliest possible withdrawal date and the latest possible withdrawal date have already been carried out, and did not significantly change the results of Kaplan-Meier estimates.

- - 1. *Outcomes and definition of outcomes in the MEGA study*

In the MEGA follow-up study, 4731 patients were included for follow-up. 1002 were lost to follow-up, 892 had a recurrence (both certain and uncertain). Mean age was 48 years, 54% of the patients were women. Mean duration of follow-up was 5.0 years when follow-up started after discontinuation of anticoagulation treatment- translating in to 20031 patient years follow-up. 79% (n=3729) patients had a compete follow-up. There were 972 recurrences, 114 occurred during anticoagulation. 673 of these recurrent events were certain.

Within men with a first idiopathic VTE (n=1039) there were 248 recurrences in 5205 follow-up years.

- 1. *Confounding factors and selection bias*

There is one main known confounder in the relation between HIV and recurrent VTE: sex. In the SHM cohort, 80% of index events are in males. Meta-analyses of multiple cohorts studying recurrence risk have shown a robust association between male sex and an elevated risk of recurrent venous thrombosis[16]. Because of the small number of index events in women, assessment of recurrence risk will only be done in men in the SHM cohort vs. control cohort (see statistical methods).

Secondly, we will perform several subgroup analyses, categorized by sex, DVT/PE (first event) and unprovoked/provoked (first event). For the latter subgroup category, the similar definitions will be made for both SHM and MEGA cohorts, to allow a valid comparison between both databases. We defined a provoked VTE in the SHM and MEGA cohort as a following exposure to the following risk factors: pregnancy, puerperium (<6 weeks after pregnancy), trauma or major surgery (< 90 days before VTE), being bed ridden for at least three days (<90 days before VTE), immobilization of a lower extremity due to a plaster cast (<90 days before VTE), use of oral contraceptives and active cancer. Active cancer is defined as: having a palliative or curative treatment for cancer at time of diagnosis of VTE, or within the previous 6 months before VTE diagnosis, or getting the diagnosis of cancer within 6 months after the otherwise unprovoked VTE.

The abovementioned variables are known to be associated with a low risk of recurrent VTE after withdrawal of anticoagulant therapy. In the MEGA study, VTEs occurring within 3 months after a hospitalization were originally adjudicated as a provoked VTE. However, studies have shown that this transient risk factor is not clearly associated with a low risk of recurrence after withdrawal of anticoagulant therapy[17]. Furthermore, in the SHM cohort we found that many index events in the SHM cohort were closely related to hospitalization, but that it wasn’t clear whether these patients had been bedridden during medical hospitalization. Given the unclear prognosis of hospitalization, our main analysis will consider a VTE in which hospitalization is the only associated provoking factor as an unprovoked VTE. We will perform a sensitivity analysis in which hospitalization is adjudicated as a provoked VTE to assess whether this alters our findings.

1. **Statistical analysis**
   1. *Methods*
      1. We will assess incidence rates for recurrent VTE in both cohorts by dividing the number of outcomes as defined above by the observed person time (n events per 1000 person years) plus their 95% confidence intervals. We will use Kaplan-Meier estimates to assess cumulative incidence estimated over the observation time. In the SHM cohort, approximately 10% of the cases are censored due to death. This degree of competing risk will probably not bias Kaplan-Meier estimates significantly. However, we will check this assumption by analyzing the cumulative incidence estimates using competing risk analysis as HIV patients may have a greater competing risk by death compared to the MEGA cohort.
      2. We will stratify analyses by sex and classification of unprovoked/provoked VTE. Due to the limited number of index events in females in the SHM cohort, our main focus will be on the VTE recurrence risk in men between the two cohorts.
      3. In our analysis of first VTE, there were strong associations between uncontrolled infection (viremia), immune deficiency (CD4 counts below 500, but also CD4/CD8 ratios <1) and VTE. The incidence of VTE was very comparable to the VTE incidence in the general population (1/1.000 person years) when CD4 counts were above 500. Therefore, we will explore whether virological status/immune status influences recurrence risk within the HIV population. In our analysis of first VTE, there were strong associations between uncontrolled infection (viremia), immune deficiency (CD4 counts below 500, but also CD4/CD8 ratios <1) on the one hand and VTE. The incidence of VTE was very comparable to the VTE incidence in the general population (1/1.000 person years) when CD4 counts were above 500. We will use Cox regression to assess hazard ratios for recurrent VTE associated with the following variables:
         - CD4 at index event
         - CD4 at withdrawal of anticoagulation
         - Viral load at index event
         - Viral load at withdrawal of anticoagulation
         - Immune response (delta CD4) between index event and withdrawal of anticoagulation.
   2. *Power considerations*

As stated before, our main analysis will concern a comparison between men with unprovoked VTE in the HIV and MEGA cohort. For calculating the power of a log-rank test, PASS software demands input of accrual time, additional follow-up after stopping accrual and drop-out rates. At current definitions, the SHM cohort has 95 male unprovoked VTE cases with a mean follow up of 5.5 years. This cohort could be described as having 11 years of accrual with no additional follow-up time (start follow-up and enrollment in 2003, end of follow-up and enrollment in 2014). For the MEGA follow-up study, follow up was a mean of 5.1 years. Patients were enrolled starting in 1999 through 2004 and response questionnaires sent in 2008 and 2009. This cohort could be described as population consisting of 5 years accrual and 5 years additional follow-up.

Considering the combined cohort, we used the following PASS input for follow-up: 4 years accrual with additional 4 years of follow-up after accrual was stopped. A cohort with such characteristics and no drop out would have a mean follow-up of 6 years. If 20% of patients drop-out (see incomplete follow-up rate MEGA cohort), then mean follow-up would be 4.8 years, which is a slight underestimation compared to the original cohorts. We assumed a failure rate of 30% at 5 years when the index date is withdrawal of anticoagulant therapy (recurrence rate with diagnosis date is the index date was 4.8 per 100 person years- at five years, this yields a rate of 23.8. We assumed that the five year Kaplan-Meier failure rate will therefore be close to 30% due to censored observations and because we will use date of anticoagulant withdrawal as index date).

So for the power calculation input we assumed a MEGA/HIV ratio of 10, accrual 4 years, additional follow up 4 years, drop-out rate 20%, failure rate 30% at five years. The minimum detectable Hazard ratios at Beta = 0,20 is 1,79. The actual power/minimally detectable hazard ratio is probably more favorable because we entered slightly conservative follow-up times.


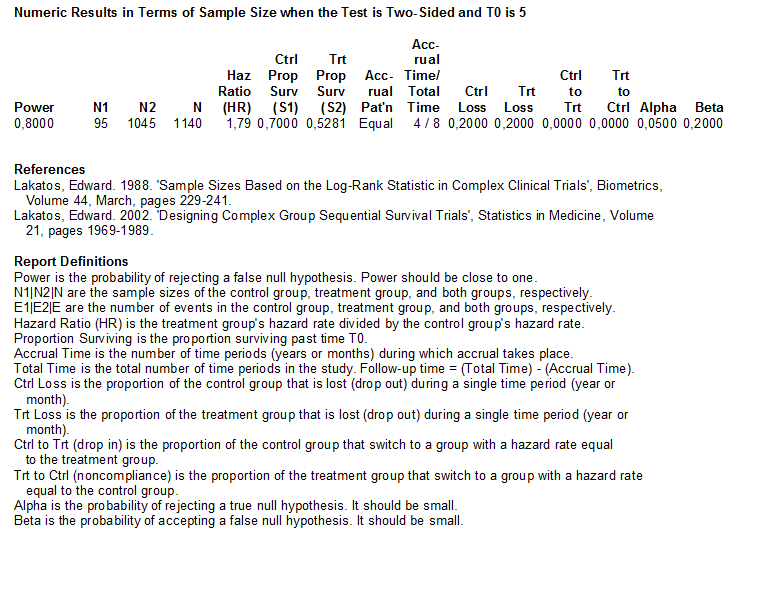
PASS 14 output:

**Tables**

- 1. *General characteristics table*

| Table 1. Baseline characteristics of participants of both cohorts with a history of a first VTE | | | | | |
| --- | --- | --- | --- | --- | --- |
|  |  | SHM cohort | | MEGA cohort | |
|  |  | *n or median* | *(%) or IQR* | *n or median* | *(%) or IQR* |
| Total N | |  |  |  |  |
| Age at start of FU (years) | |  |  |  |  |
| Male sex | |  |  |  |  |
| Descent | |  |  |  |  |
|  | Caucasian |  |  |  |  |
|  | African |  |  |  |  |
|  | Asian |  |  |  |  |
| Observation time (months) | |  |  |  |  |
| Idiopathic first VTE | |  |  |  |  |
| Provoked first VTE | |  |  |  |  |
| DVT only | |  |  |  |  |
| PE with or without DVT | |  |  |  |  |
| Duration of anticoagulation (months) | | |  |  |  |
| VTE, venous thromboembolism; FU, follow-up; DVT, deep vein thrombosis; PE, pulmonary embolism. | | | | | |

- 1. *Planned table(s)*

| Table 2. Numbers and absolute risks of recurrent VTE according to characteristics of the first VTE and HR for HIV-1 infection | | | | | | | | | | | | |  |
| --- | --- | --- | --- | --- | --- | --- | --- | --- | --- | --- | --- | --- | --- |
|  |  | SHM cohort | | |  | MEGA cohort | | |  |  |  |  |  |
|  |  | *Events* | *Observation* | *IR** |  | *Events* | *Observation* | *IR** |  | *IRR*† | *95%CI* | *IRR*‡ | *95%CI* |
|  |  | *(n)* | *time (years)* |  |  | *(n)* | *time (years)* |  |  |  |  |  |  |
| Any first VTE | |  |  |  |  |  |  |  |  |  |  |  |  |
| Sex | |  |  |  |  |  |  |  |  |  |  |  |  |
|  | Males |  |  |  |  |  |  |  |  |  |  |  |  |
|  | Females |  |  |  |  |  |  |  |  |  |  |  |  |
| Idiopathic VTE | |  |  |  |  |  |  |  |  |  |  |  |  |
| Provoked VTE | |  |  |  |  |  |  |  |  |  |  |  |  |
| Deep vein thrombosis (DVT) | | |  |  |  |  |  |  |  |  |  |  |  |
| PE, with or without DVT | |  |  |  |  |  |  |  |  |  |  |  |  |
| VTE, venous thrombotic event; IRR, incidence rate ratio; IR, incidence rate; CI, confidence interval; PE, pulmonary embolism; | | | | | | | | | | |  |  |  |
| * per 1000 person-years; † Adjusted for age and sex; ‡ Adjusted for age, sex, co-infections | | | | | | |  |  |  |  |  |  |  |

| Table 3. Hazard ratios for recurrent VTE according to recent exposure* by listed risk factors | | | | | | | | |  |  |  |  |  |
| --- | --- | --- | --- | --- | --- | --- | --- | --- | --- | --- | --- | --- | --- |
|  |  | *Baseline* | | |  | *Risk period* | | |  |  |  |  |  |
|  |  | *Events* | *Observation* | *IR*† |  | *Events* | *Observation* | *IR*† |  | *HR* | *95%CI* | *HR*‡ | *95%CI* |
|  |  | *(n)* | *time (years)* |  |  | *(n)* | *time (years)* |  |  |  |  |  |  |
| PI started or stopped | |  |  |  |  |  |  |  |  |  |  |  |  |
| Abacavir started or stopped | |  |  |  |  |  |  |  |  |  |  |  |  |
| Co-infections | |  |  |  |  |  |  |  |  |  |  |  |  |
| Therapy failure§ | |  |  |  |  |  |  |  |  |  |  |  |  |
| VTE, venous thrombotic event; HR, hazard ratio; CI, confidence interval; PI, protease inhibitor; STD, sexual transmitted disease; | | | | | | | | | | |  |  |  |
| cART, combined antiretroviral therapy; * risk period was defined as within the previous 3 months before event; † IR, incidence rate | | | | | | | | | | |  |  |  |
| per 1000 person-years; ‡ Adjusted for age and sex; § Detectable HIV-RNA after being undetectable or >15% decrease in CD4/CD8 ratio. | | | | | | | | | | | |  |  |

1. **Reference list**

1 Matta F, Yaekoub AY, Stein PD. Human immunodeficiency virus infection and risk of venous thromboembolism. *Am J Med Sci* 2008; **336**: 402-6.

2 Rasmussen LD, Dybdal M, Gerstoft J, Kronborg G, Larsen CS, Pedersen C, Pedersen G, Jensen J, Pedersen L, Sorensen HT, Obel N. HIV and risk of venous thromboembolism: a Danish nationwide population-based cohort study. *HIV Med* 2011; **12**: 202-10.

3 Auerbach E, Aboulafia DM. Venous and arterial thromboembolic complications associated with HIV infection and highly active antiretroviral therapy. *Semin Thromb Hemost* 2012; **38**: 830-8.

4 Malek J, Rogers R, Kufera J, Hirshon JM. Venous thromboembolic disease in the HIV-infected patient. *The American journal of emergency medicine* 2011; **29**: 278-82.

5 Asherson RA, Gomez-Puerta JA, Marinopoulos G. Recurrent pulmonary thromboembolism in a patient with systemic lupus erythematosus and HIV-1 infection associated with the presence of antibodies to prothrombin: a case report. *Clin Infect Dis* 2005; **41**: e89-92.

6 de Gaetano Donati K, Tacconelli E, Scoppettuolo G, De Stefano V, Cauda R, Tumbarello M. Recurrent venous thrombosis in a patient with haemophilia A and HIV infection. *Haematologica* 2002; **87**: ECR04.

7 Dos Santos VM, Teles LT, Leao CE, Lopes JW, Fastudo CA, Lima RL. Recurrent deep venous thrombosis in an HIV-positive and injecting drug user woman. *Indian J Sex Transm Dis* 2012; **33**: 41-3.

8 Shah I. Recurrent thrombosis in an HIV-1 infected child. *Indian J Sex Transm Dis* 2012; **33**: 121-3.

9 Jong E, Louw S, Meijers JC, de Kruif MD, ten Cate H, Buller HR, Mulder JW, van Gorp EC. The hemostatic balance in HIV-infected patients with and without antiretroviral therapy: partial restoration with antiretroviral therapy. *AIDS Patient Care STDS* 2009; **23**: 1001-7.

10 Jong E, Meijers JC, van Gorp EC, Spek CA, Mulder JW. Markers of inflammation and coagulation indicate a prothrombotic state in HIV-infected patients with long-term use of antiretroviral therapy with or without abacavir. *AIDS Res Ther* 2010; **7**: 9.

11 Jong E, Louw S, van Gorp EC, Meijers JC, ten Cate H, Jacobson BF. The effect of initiating combined antiretroviral therapy on endothelial cell activation and coagulation markers in South African HIV-infected individuals. *Thromb Haemost* 2010; **104**: 1228-34.

12 Bezemer ID, van der Meer FJ, Eikenboom JC, Rosendaal FR, Doggen CJ. The value of family history as a risk indicator for venous thrombosis. *Arch Intern Med* 2009; **169**: 610-5.

13 Blom JW, Doggen CJ, Osanto S, Rosendaal FR. Malignancies, prothrombotic mutations, and the risk of venous thrombosis. *JAMA* 2005; **293**: 715-22.

14 Flinterman LE, van Hylckama Vlieg A, Rosendaal FR, Cannegieter SC. Body height, mobility, and risk of first and recurrent venous thrombosis. *J Thromb Haemost* 2015; **13**: 548-54.

15 Flinterman LE, van Hylckama Vlieg A, Cannegieter SC, Rosendaal FR. Long-Term Survival in a Large Cohort of Patients with Venous Thrombosis: Incidence and Predictors. *Plos Med* 2012; **9**: e1001155.
16 Douketis J, Tosetto A, Marcucci M, et al. Risk of recurrence after venous thromboembolism in men and women: patient level meta-analysis. *BMJ*. 2011;342(7796):d813
17 Bjøri E, Arshad N, Johnsen HS, Hansen J-B, Brækkan SK. Hospital-related first venous thromboembolism and risk of recurrence. J Thromb Haemost 2016; 14: 2368–75.

1. **Appendix 1: case finding and adjudication events SHM cohort**

All patients in the SHM database that had received any of these anticoagulants since January 2003 were potential patients who might have experienced a HIV-associated VTE. Potential VTE cases were identified in the SHM database using the following search-criteria:

- All HIV-infected patients on coumarines or new oral anticoagulants (NOACs), regardless of duration
- All HIV-infected patients on LMWH/heparin for ≥2 weeks
- All HIV-infected patients who received the combination of LWMH/heparin and coumarines at any time together
- All HIV-infected patients who received thrombolytic therapy
- All HIV-infected patients that were deceased with any of the following registered as the cause of death: a) a VTE related death, b) a sudden death or c) patients that were admitted to the hospital for VTE and died without a reported cause of death

Patients identified by these search-criteria were studied case-by-case on the occurrence of HIV-associated VTE by cooperative efforts, including querying the local SHM data-manager, and by checking **all** local medical records of identified patients by the research team. By case-by-case evaluation, we were able to identify the patients that used anticoagulants for VTE, or for other reasons (e.g. atrial fibrillation). VTE was defined by any of the following events: thrombophlebitis, calf vein thrombosis or “distal DVT”, deep vein thrombosis or “proximal DVT” , pulmonary embolism, splanchnic vein thrombosis, portal vein thrombosis, cerebral venous sinus thrombosis, arm vein thrombosis.

A VTE was considered as confirmed when diagnosed by: ultrasound, venography, echocardiography, ventilation/perfusion lung scan, computed tomography scan, autopsy verified VTE, VTE was registered as the (probable) cause of death.

VTEs occurring prior to HIV diagnosis were be evaluated as follows:

- VTEs within 6 months prior to HIV diagnosis were recorded as VTE related to HIV regardless of CD4 count at HIV diagnosis, except if acute HIV was diagnosed. An acute HIV after the VTE event is identified by the documentation of acute antiretroviral syndrome in the medical record or by a negative/indeterminate results of the p31 western blot.

-VTE within 12 months prior to HIV diagnosis was recorded as a VTE related to HIV if CD4 count was <200 cell/mm^3^ at HIV diagnosis.

- All other VTE’s that were reported in medical correspondence, either as a medical diagnosis in a letter or in the medical history, and having occurred at least 12 months before the diagnosis of HIV and not fulfilling the third criterion described above, were considered non HIV-associated VTE’s. However, only those that were clearly described as provoked or unprovoked were included in our database.

We have evaluated the sensitivity and accurateness of our case finding strategy in two pilot studies in EMC and UMCG separately. In the EMC, all HIV patients have been screened case by case by the investigators on VTE occurrence. 44 VTE cases were identified and represented the golden standard. By searching the SHM database by 1 of the criteria (any coumarines exposure) of our proposed search strategy, we were able to retrieve 39 of 44 patients (89%) correctly out of a total yield of 100 patients that used coumarines. When we considered events which occurred after 2003 (date after which SHM started to systematically collect use of anticoagulants), sensitivity was 100%

In the UMCG, we checked if any cases were being missed by cross-referencing our findings with two sources: 1) an existing HIV research database in which patients thrombotic phenotype was included (eleven VTE cases) and 2) a list of VTE patients provided by the HIV care team (six cases). In the UMCG, 53 patients had registered coumarine use. These methods of cross-referencing gave a sensitivity of respectively (9/11) 82% and (5/6) 83%. However, the cases missed in these methods were of questionable clinical significance: one event took place before HIV infection, another case had lemierre’s syndrome, for which only antibiotic and not anticoagulant therapy was prescribed. Based on these 2 pilots, the search sensitivity appears to be at least 80%, which we considered the minimum sensitivity to successfully use this search strategy on the SHM database. Considering events which are relevant to our current study (recurrent VTE after a DVT of the legs and/or PE) our sensitivity seems to be 100%.
