# Supplementary material for: Risk of recurrent venous thromboembolism in patients with HIV infection: A nationwide cohort study
Source: PLoS Med. 2020 May 14;17(5):e1003101. doi: 10.1371/journal.pmed.1003101 (PMC7224453; doi:10.1371/journal.pmed.1003101)
Supplement: S1 Supplementary Methods — (DOCX) [file pmed.1003101.s003.docx]

**Supplementary methods and metadata**

**List of participating centers**

**Participating centers with corresponding numbers on map (N=12)**

3 Amsterdam UMC, AMC site, Amsterdam **academic**

5 Onze Lieve Vrouwe Gasthuis, Amsterdam

6 Medisch Centrum Slotervaart, Amsterdam

9 Rijnstate Ziekenhuis, Arnhem

10 HAGA Ziekenhuis, The Hague

11 Haaglanden Medical Centre (HMC), The Hague

15 University Medical Centre Groningen, Groningen **academic**

18 Leiden University Medical Centre, Leiden **academic**

20 Maastricht University Medical Centre, Maastricht **academic**

22: Erasmus Medical Centre, Rotterdam **academic**

23 Maasstad Ziekenhuis, Rotterdam

24 Elisabeth-Tweesteden Ziekenhuis, Tilburg

**ATC-codes used in ATHENA for detecting potential VTE cases**

| A case was flagged as a potential VTE if any if the following medications were registered to a patient, irrespective of registered duration of treatment: | | |
| --- | --- | --- |
| 481 | Fenprocoumon | Fenprocoumon |
| 481 | Marcoumar® | Fenprocoumon |
| 227 | Sintrom® | Acenocoumarol |
| 249 | Alteplase | Alteplase |
| 267 | Anistreplase | Anistreplase |
| 855 | Medacinase® | Urokinase |
| 821 | Metalyse® | Tenecteplase |
| 758 | Rapilysin® | Reteplase |
| 758 | Reteplase | Reteplase |
| 801 | Streptase® | Streptokinase |
| 801 | Streptokinase | Streptokinase |
| 821 | Tenecteplase | Tenecteplase |
| 855 | Urokinase | Urokinase |
| For the following medication, a potential VTE case was flagged if use was registered for at least 14 days: | | |
| 503 | Arixtra® | Fondaparinux |
| 437 | Clexane® | Enoxaparin |
| 392 | Dalteparine | Dalteparin |
| 393 | Danaparoïde | Danaparoid |
| 437 | Enoxaparin | Enoxaparin |
| 503 | Fondaparinux | Fondaparinux |
| 392 | Fragmin® | Dalteparin |
| 653 | Fraxiparine® | Nadroparin |
| 653 | Fraxodi® | Nadroparin |
| 833 | Innohep® | Tinzaparin |
| 653 | Nadroparin | Nadroparin |
| 393 | Orgaran® | Danaparoid |
| 833 | Tinzaparin | Tinzaparin |
| 525 | Heparinoids | Heparinoids |

**Decision rules for recurrent VTE**

To be classified as a certain recurrence, a reported recurrence should fulfil one of the following criteria.

1. A discharge letter was present concluding a diagnosis of recurrence, based on available clinical and radiological data. This recurrence should be in a different vein or in a different part of the body than the first event. The discharge letter had to contain information about instrumental diagnostic procedures. If location of either first or second thrombosis was not known or was similar to the first event, an event was still classified as certain if at least three months had passed since the first thrombosis.
2. A discharge letter was not available (e.g. when treating physician was unknown) but both the anticoagulation clinic and the patient reported a recurrence at a clearly different location than the first event (contralateral leg, DVT after PE or vice versa) or a time period of more than a year had passed between the two events.
3. A registered cause of death from PE or DVT at least six months after the first event.

Possible recurrences were defined by four criteria, one of which had to apply:

1. A diagnosis of a possible recurrence in the discharge letter, where clinical and radiological data could not distinguish between an extension of the first and a new thrombotic event.
2. A discharge letter was not available but both the patient and the anticoagulation clinic reported a recurrence within a year after the first event.
3. Information was only available from either the patient or the anticoagulation clinic.
4. A registered cause of death from PE or DVT within six months after the first event.

**Full listing of participating staff in the ATHENA cohort**

The ATHENA cohort is managed by Stichting HIV Monitoring and supported by a grant from the Dutch Ministry of Health, Welfare and Sport through the Centre for Infectious Disease Control of the National Institute for Public Health and the Environment.

**CLINICAL CENTRES**

** denotes site coordinating physician*

**Amsterdam UMC, AMC site, Amsterdam:** *HIV treating physicians:* M. van der Valk*, S.E. Geerlings, A. Goorhuis, J.W. Hovius, B. Lempkes, F.J.B. Nellen, T. van der Poll, J.M. Prins, P. Reiss, M. van Vugt, W.J. Wiersinga, F.W.M.N. Wit. *HIV nurse consultants:* M. van Duinen, J. van Eden, A. Hazenberg, A.M.H. van Hes, F.J.J. Pijnappel, S.Y. Smalhout, A.M. Weijsenfeld. *HIV clinical virologists/chemists:* S. Jurriaans, N.K.T. Back, H.L. Zaaijer, B. Berkhout, M.T.E. Cornelissen, C.J. Schinkel, K.C. Wolthers. **Amsterdam UMC,** **VUmc site, Amsterdam:** *HIV treating physicians:* E.J.G. Peters*, M.A. van Agtmael, M. Bomers, K.C.E. Sigaloff. *HIV nurse consultants:* M. Heitmuller, L.M. Laan. *HIV clinical virologists/chemists:* C.W. Ang, R. van Houdt, M. Jonges, A.M. Pettersson, J. van Prehn. **Admiraal De Ruyter Ziekenhuis, Goes:** *HIV treating physicians:* M. van den Berge, A. Stegeman. *HIV nurse consultants:* S. Baas, L. Hage de Looff. *HIV clinical virologists/chemists:* B. Wintermans, J. Veenemans. **Catharina Ziekenhuis, Eindhoven:** *HIV treating physicians:* M.J.H. Pronk*, H.S.M. Ammerlaan. *HIV nurse consultants:* E.S. de Munnik. *HIV clinical virologists/chemists:* A.R. Jansz, J. Tjhie, M.C.A. Wegdam, B. Deiman, V. Scharnhorst. **DC Klinieken Lairesse - Hiv Focus Centrum:** *HIV treating physicians:* A. van Eeden*, M. van der Valk. *HIV nurse consultants:* W. Brokking, L.J.M. Elsenburg, H. Nobel. *HIV clinical virologists/chemists:* M. Damen. **ETZ (Elisabeth-TweeSteden Ziekenhuis), Tilburg:** *HIV treating physicians:* M.E.E. van Kasteren*, M.A.H. Berrevoets, A.E. Brouwer. *HIV nurse consultants:* A. Adams, B.A.F.M. de Kruijf-van de Wiel, S. Keelan-Pfaf, B. van de Ven. *Data collection:* B.A.F.M. de Kruijf-van de Wiel, B. van der Ven. *HIV clinical virologists/chemists:* A.G.M. Buiting, J.L. Murck, D. Versteeg. **Erasmus MC, Rotterdam:** *HIV treating physicians:* M.E. van der Ende*, H.I. Bax, E.C.M. van Gorp, J.L. Nouwen, B.J.A. Rijnders, C.A.M. Schurink, A. Verbon, T.E.M.S. de Vries-Sluijs, N.C. de Jong-Peltenburg. *HIV nurse consultants:* N. Bassant, J.E.A. van Beek, M. Vriesde, L.M. van Zonneveld. *Data collection:* H.J. van den Berg-Cameron, J. de Groot. *HIV clinical virologists/chemists:* C.A.B. Boucher, M.P.G Koopmans, J.J.A van Kampen. **Flevoziekenhuis, Almere:** *HIV treating physicians:* J. Branger*, R.A. Douma. *HIV nurse consultant:* C.J.H.M. Duijf-van de Ven. **HagaZiekenhuis, Den Haag:** *HIV treating physicians:* E.F. Schippers*, C. van Nieuwkoop. *HIV nurse consultants:* J.M. van IJperen, J. Geilings. *Data collection:* G. van der Hut. *HIV clinical virologist/chemist:* N.D. van Burgel. **HMC (Haaglanden Medisch Centrum), Den Haag:** *HIV treating physicians:* E.M.S. Leyten*, L.B.S. Gelinck, F. Mollema. *HIV nurse consultants:* S. Davids-Veldhuis, A.Y. van Hartingsveld, G.S. Wildenbeest. *HIV clinical virologists/chemists:* E. Heikens. **Isala, Zwolle:** *HIV treating physicians:* P.H.P. Groeneveld*, J.W. Bouwhuis, A.J.J. Lammers. *HIV nurse consultants:* S. Kraan, A.G.W. van Hulzen, M.S.M. Kruiper. *Data collection:* G.L. van der Bliek, P.C.J. Bor. *HIV clinical virologists/chemists:* P. Bloembergen, M.J.H.M. Wolfhagen, G.J.H.M. Ruijs. **Leids Universitair Medisch Centrum, Leiden:** *HIV treating physicians:* F.P. Kroon*, M.G.J. de Boer, H. Scheper, H. Jolink. *HIV nurse consultants:* W. Dorama, N. van Holten. *HIV clinical virologists/chemists:* E.C.J. Claas, E. Wessels. **Maasstad Ziekenhuis, Rotterdam:** *HIV treating physicians:* J.G. den Hollander*, K. Pogany, A. Roukens, R. Douma. *HIV nurse consultants:* M. Kastelijns, J.V. Smit, E. Smit, D. Struik-Kalkman, C. Tearno. *Data collection:* T. van Niekerk. *HIV clinical virologists/chemists:* O. Pontesilli. **Maastricht UMC+, Maastricht:** *HIV treating physicians:* S.H. Lowe*, A.M.L. Oude Lashof, D. Posthouwer. *HIV nurse consultants:* R.P. Ackens, K. Burgers, J. Schippers. *Data collection:* B. Weijenberg-Maes. *HIV clinical virologists/chemists:* I.H.M. van Loo, T.R.A. Havenith. **MC Slotervaart, Amsterdam:** *HIV treating physicians:* J.W. Mulder*, S.M.E. Vrouenraets, F.N. Lauw. *HIV nurse consultants:* M.C. van Broekhuizen, D.J. Vlasblom, M. Kroeze. *HIV clinical virologists/chemists:* P.H.M. Smits. **MC Zuiderzee, Lelystad:** *HIV treating physicians:* S. Weijer*, R. El Moussaoui. *HIV nurse consultant:* A.S. Bosma. **Medisch Centrum Leeuwarden, Leeuwarden:** *HIV treating physicians:* M.G.A.van Vonderen*, L.M. Kampschreur. *HIV nurse consultants:* S. Faber, R. Steeman-Bouma. *HIV clinical virologists/chemists:* J Weel. **Medisch Spectrum Twente, Enschede:** *HIV treating physicians:* G.J. Kootstra*, C.E. Delsing. *HIV nurse consultants:* M. van der Burg-van de Plas, H. Heins. **Noordwest Ziekenhuisgroep, Alkmaar:** *HIV treating physicians:* W. Kortmann*, G. van Twillert*, R. Renckens. *HIV nurse consultant and data collection:* D. Ruiter-Pronk, F.A. van Truijen-Oud. *HIV clinical virologists/chemists:* J.W.T. Cohen Stuart, ER. Jansen, M. Hoogewerf, W. Rozemeijer, W. A. van der Reijden, J.C. Sinnige. **OLVG, Amsterdam:** *HIV treating physicians:* K. Brinkman*, G.E.L. van den Berk, W.L. Blok, P.H.J. Frissen, K.D. Lettinga W.E.M. Schouten, J. Veenstra. *HIV nurse consultants:* C.J. Brouwer, G.F. Geerders, K. Hoeksema, M.J. Kleene, M. Knapen, I.B. van der Meché, E. Mulder-Seeleman, A.J.M. Toonen, S. Wijnands. *HIV clinical virologists:* D. Kwa. **Radboudumc, Nijmegen:** *HIV treating physicians:* R. van Crevel*, A.S.M. Dofferhoff, H.J.M. ter Hofstede, J. Hoogerwerf, M. Keuter, O. Richel. *HIV nurse consultants:*M. Albers, K.J.T. Grintjes-Huisman, M. de Haan, M. Marneef, R. Strik-Albers. *HIV clinical virologists/chemists:*J. Rahamat-Langendoen, F.F. Stelma. *HIV clinical pharmacology consultant:*D. Burger. **Rijnstate, Arnhem:** *HIV treating physicians:* E.H. Gisolf*, R.J. Hassing, M. Claassen. *HIV nurse consultants:* G. ter Beest, P.H.M. van Bentum, N. Langebeek. *HIV clinical virologists/chemists:* R. Tiemessen, C.M.A. Swanink. **Spaarne Gasthuis, Haarlem:** *HIV treating physicians:* S.F.L. van Lelyveld*, R. Soetekouw. *HIV nurse consultants:* L.M.M. van der Prijt, J. van der Swaluw. *Data collection:* N. Bermon. *HIV clinical virologists/chemists:* W.A. van der Reijden, R. Jansen, B.L. Herpers, D.Veenendaal. **Medisch Centrum Jan van Goyen, Amsterdam:** *HIV treating physicians:* D.W.M. Verhagen. *HIV nurse consultants:* M. van Wijk. **Universitair Medisch Centrum Groningen, Groningen:** *HIV treating physicians:* W.F.W. Bierman*, M. Bakker, J. Kleinnijenhuis, E. Kloeze, A. Middel, Y. Stienstra, C.L. Vermont, KM. Wouthuyzen-Bakker. *HIV nurse consultants:* A. Boonstra, H. de Groot-de Jonge, P.A. van der Meulen, D.A. de Weerd. *HIV clinical virologists/chemists:* H.G.M. Niesters, C.C. van Leer-Buter, M. Knoester. **Universitair Medisch Centrum Utrecht, Utrecht:** *HIV treating physicians:* A.I.M. Hoepelman*, J.E. Arends, R.E. Barth, A.H.W. Bruns, P.M. Ellerbroek, T. Mudrikova, J.J. Oosterheert, M.J.A. de Regt, E.M. Schadd, M.W.M. Wassenberg, M.A.D. van Zoelen. *HIV nurse consultants:* K. Aarsman, B.M.G. Griffioen-van Santen, I. de Kroon, C.S.A.M. van Rooijen. *Data collection:* M. van Berkel, C.S.A.M. van Rooijen. *HIV clinical virologists/chemists:* R. Schuurman, F. Verduyn-Lunel, A.M.J. Wensing.

**COORDINATING CENTRE**

*Director:* P. Reiss. *Deputy director:* S. Zaheri. *Data analysis:* D.O. Bezemer, A.I. van Sighem, C. Smit, F.W.M.N. Wit. *Data management and quality control:* M. Hillebregt, A. de Jong, T. Woudstra. *Data monitoring:* D. Bergsma, S. Grivell, R. Meijering, M. Raethke, T. Rutkens. *Data collection:* L. de Groot, M. van den Akker, Y. Bakker, A. El Berkaoui, M. Bezemer, N. Brétin, E. Djoechro, J. Geerlinks, E. Kruijne, C. Lodewijk, E. Lucas, R. van der Meer, L. Munjishvili, F. Paling, B. Peeck, C. Ree, R. Regtop, Y. Ruijs, L. van de Sande, M. Schoorl, P. Schnörr, E. Tuijn, L. Veenenberg, S. van der Vliet, E.C. Witte. *Patient registration:* B. Tuk.
