# Supplementary material for: Risk of recurrent venous thromboembolism in patients with HIV infection: A nationwide cohort study
Source: PLoS Med. 2020 May 14;17(5):e1003101. doi: 10.1371/journal.pmed.1003101 (PMC7224453; doi:10.1371/journal.pmed.1003101)
Supplement: S1 Supplementary Analyses — (DOCX) [file pmed.1003101.s004.docx]

Supplementary analyses

**Analyses performed after peer review**

One reviewer expressed concern that the results of a higher risk of VTE may have been driven by participants with an initial detectable viral load. Therefore, we reran model excluding these participants. Models adjusted for age, sex and unprovoked/provoked index VTE

|  | **HR (95%CI) Original model*** | **HR (95%CI) only virally suppressed at index VTE** |
| --- | --- | --- |
| Overall | 1.16 (0.84-1.62) | 1.33 (0.89-1.99) |
| Follow-up < 6 years | 1.22 (0.87-1.73) | 1.32 (0.86-2.01) |
| Follow-up < 1 year | 1.67 (1.04-2.70) | 1.64 (0.89 -3.04) |
| Follow up 1-6 years | 0.94 (0.58-1.55) | 0.94 (0.53-1.68) |

One additional analysis was inferred by concerns from peer reviewers about period effects: the fact that the cohorts had followed-up patients over different time periods. Besides simply removing subsegmental PE, the influence of period effects was additionally explored by choosing overlapping periods of follow-up. Ideally, we would choose overlap in index dates *and* overlap in follow-up after withdrawal of anticoagulation. However, there is only overlap between the two cohorts in index VTEs for two years (2003-2004), rendering such an approach futile as only 12% of the participants in the ATHENA cohort had their index event in these years. Therefore, we included MEGA participants who had their index event in 2003 or later (n=858) and limited follow-up time in ATHENA participants to the 31^st^ of December 2010 (n=87), excluding people with index events after this date. We reiterate that in the context of our conclusions this should be acceptable as we are mainly concerned in overascertainment of the outcome, not in overascertainment of index VTE. Also, we can reasonably assume smaller changes in diagnostic practice between 2004-2009 as opposed to a twenty year period (1999-2018). Results from the cox model are shown below. The initial time split is abandoned as data become sparse at late follow-up for both cohorts. Models adjusted for age, sex, and unprovoked/provoked index VTE. The time split from original models is abandoned as data become sparse at late follow up inherent to the period selection (almost no patients have follow-up > 5 years).

| **Sensitivity analysis overlapping period of follow-up** | | |
| --- | --- | --- |
|  | **HR (95%CI) Original model*** | **HR (95%CI) overlapping period** |
| Overall | 1.16 (0.84-1.62) | 1.32 (0.78-2.24) |
| Follow-up < 1 year | 1.67 (1.04-2.70) | 1.93 (1.04 -3.61) |

One reviewer suggested analyzing the effect of anti-retroviral therapy initiation specifically around the time of index VTE and the influence this may have on recurrent VTE. We considered this idea interesting as this may indeed be a marker for specific improvement between index event and withdrawal of anticoagulation, with the caveat that it does not inform on the extent of recovery. In the following analysis, we defined starting ART as being ART naïve and first documented prescription of ART 1 month prior until 3 months after index VTE. The rest of the participants, including those already on ART, we considered as not exposed. 22 participants started ART within the defined time period. The model is, just as for the analysis of CD4 counts, adjusted for age, sex, infection at index VTE and unprovoked/provoked:

HR: 0.52, 95%CI 0.15-1.80

One of the peer reviewers requested to examine the characteristics of participants lost to follow-up. We restricted this to participants who were lost to follow up not due to end of study.

| **Characteristics of participants with incomplete follow-up** | | | | |
| --- | --- | --- | --- | --- |
|  | **PWH complete (n= 148)** | **PWH censored* (n=5)** | **MEGA complete (n=3,594)** | **MEGA censored* (n=411)** |
| **Male sex, n (%)** | 116 (82%) | 5 (100%) | 1,628 (45%) | 185 (45%) |
| **Age at first VTE, median years (IQR)** | 49 (42-58) | 31 (26-35) | 49 (39-58) | 48 (36-58) |
| **Location of first VTE** |  |  |  |  |
| Pulmonary embolism  (with/without DVT elsewhere), n (%) | 69 (48%) | 3 (60%) | 1473 (41%) | 164 (40%) |
| **Unprovoked first VTE, n (%)** | 100 (70%) | 3 (60%) | 1,249 (35%) | 112 (27%) |
| Undetectable viral load at index | 82 (57%) | 5 (50%) | n/a | n/a |
| CD4 median at index event (IQR) | 400  (228-600) | 178 (110-240) | n/a | n/a |
| *potentially informative censoring: transfer out, loss to follow up | | | | |
