# Supplementary figures and images for: Risk of recurrent venous thromboembolism in patients with HIV infection: A nationwide cohort study
Source: PLoS Med. 2020 May 14;17(5):e1003101. doi: 10.1371/journal.pmed.1003101 (PMC7224453; doi:10.1371/journal.pmed.1003101)

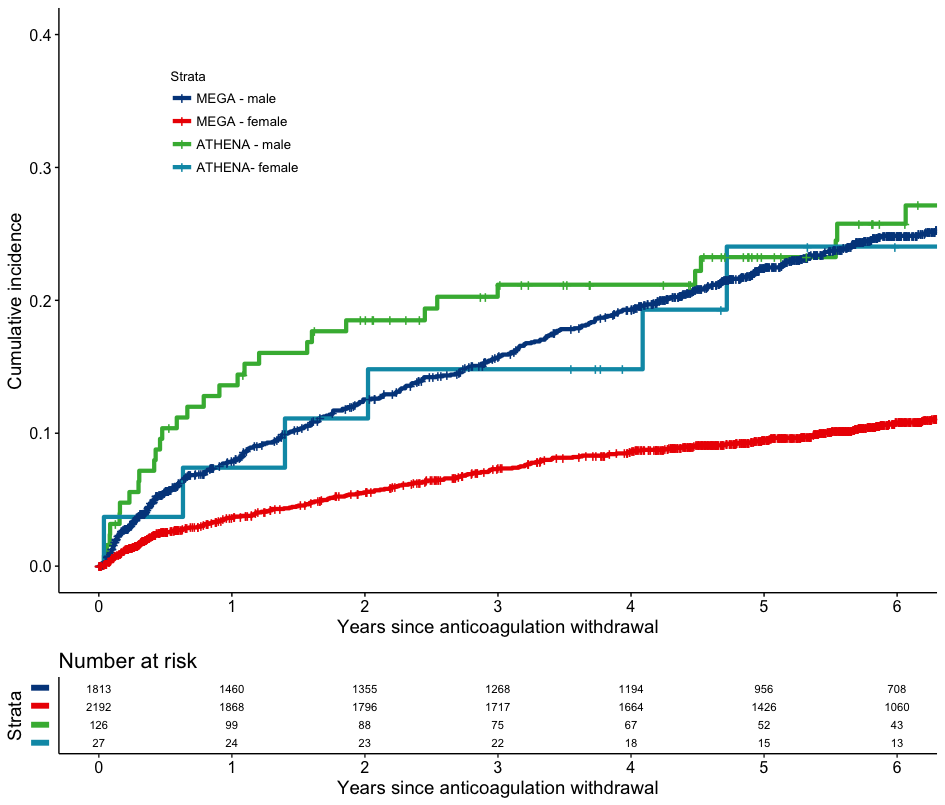

Supplement: S1 Fig — (TIF) [file pmed.1003101.s005.tif]

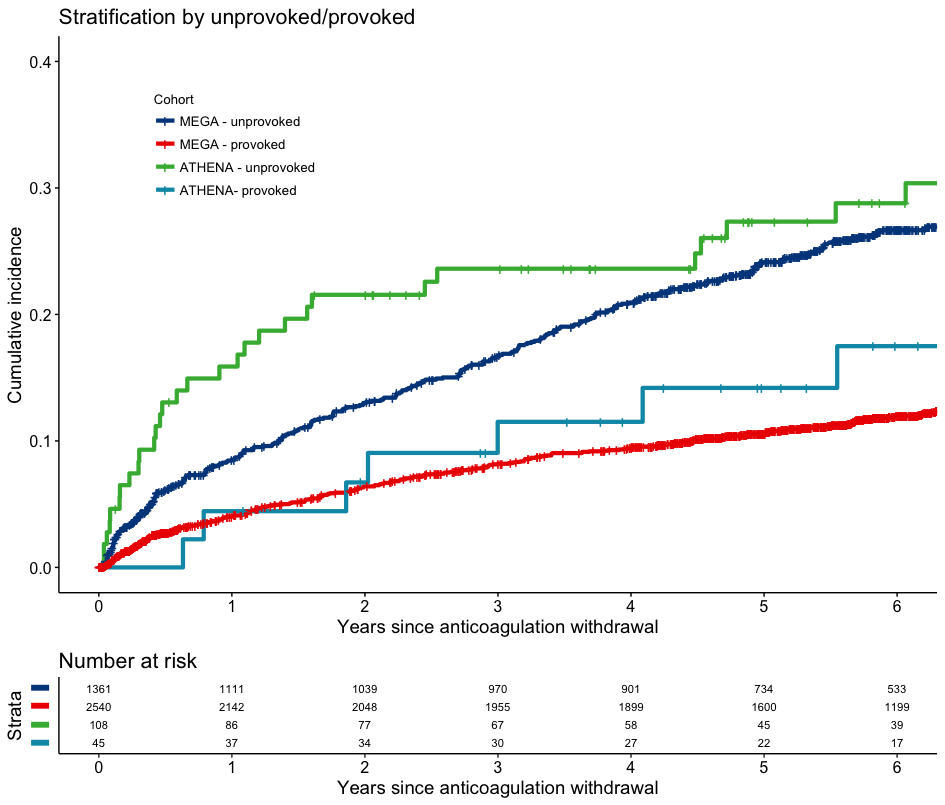

Supplement: S2 Fig — (TIF) [file pmed.1003101.s006.tif]
