# Supplementary material for: Risk of recurrent venous thromboembolism in patients with HIV infection: A nationwide cohort study
Source: PLoS Med. 2020 May 14;17(5):e1003101. doi: 10.1371/journal.pmed.1003101 (PMC7224453; doi:10.1371/journal.pmed.1003101)
Supplement: Attachment — Submitted filename: Response peer review V4.docx [file pmed.1003101.s007.docx]

**Requests from the Editors:**

1. You will note that one referee questions the validity of the study, and we suggest a detailed response on this point.

We have responded in detail to the comments of the referees, including referee 4 who had several questions about the validity of the study. Where issues were already addressed somewhat, we have endeavored to elaborate more clearly. With regards to issues surrounding different periods of analysis, we added extra sensitivity analyses and discuss these accordingly.

We hope you find that we have attempted to be frank about limitations and that we have reasoned appropriately what these mean in this specific research context. If anything, most sensitivity analyses led to a larger estimated hazard ratio in association with HIV and at the very least, findings were highly consistent with respect to the direction of effect in all of the sensitivity and/or subgroup analyses performed.

2. In your abstract and elsewhere, please add p values alongside 95% CI where available. 

We are hesitant of reporting p-values in the manuscript. A recent publication advises not to use p-values in epidemiological studies that have a null hypothesis (like ours; see Amrhein V, Greenland S, McShane B. Scientists rise up against statistical significance. Nature. 2019;567:305-307), as it may lead to ‘dichotomania’ (rejecting a null-hypothesis only on the basis of a p-value<0.05). To avoid this problem, we provide in the abstract hazard ratios with point estimates (to provide the strength of the association) together with 95%CIs to indicate their precision. P-values can be inferred from the 95%CI and addition of p-values might make the abstract and results section more ‘cluttered’.

Additionally, the main underlying clinical question is whether people living with HIV who suffer a VTE should continue anticoagulation. Within the current treatment paradigm (indefinite treatment for most cases with unprovoked VTE), it is mainly important that as a group, people living with HIV do not have an importantly lower risk of recurrent VTE. A p-value is not helpful in this respect, the 95% CI is.

3. Please add summary demographic details for study participants to the abstract.

These are now added to the abstract (page 2). It reads as follows: “There were 153 PWH (82% men, median age 48 years) and 4,005 uninfected controls (45% men, median 49 years) with a first VTE (71% unprovoked in PWH, 34% unprovoked in controls) available for analysis.”

4. Please add a new final sentence to the "methods and findings" subsection of your abstract, which should summarize the study's main limitations.

This sentence is now added. It reads as follows (line 62, page 2): “The main limitation to this study is that VTEs were adjudicated differently in the cohorts, and diagnostic practices changed during the 20-year study period. However, sensitivity analyses addressing these potential sources of bias confirmed our principal analyses.”

5. After the abstract, we will need to ask you to add a new and accessible "author summary" section in non-identical prose. You may find it helpful to refer to one or two recent research papers published in PLOS Medicine to get a sense of the preferred style. 

We have added the following author summary (page 3):

Author summary

Why was this study done?

- The human immunodeficiency virus (HIV) pandemic currently affects approximately 40 million people and causes significant morbidity, including a markedly increased risk of a first venous thromboembolism (VTE).
- The recurrence risk of VTE in people living with HIV (PWH) is unknown, while ideally this risk should guide the anticoagulant therapy duration after a first VTE.
- Our study determined recurrent VTE risk in PWH compared to uninfected controls.

What did the researchers do and find?

- We performed an observational cohort study between 1999-2018 using data from the national ATHENA PWH cohort in the Netherlands and the Dutch MEGA cohort of HIV-uninfected controls with a first VTE.
- The recurrent VTE incidence rate per 100 person-years of follow-up was higher in PWH (5.2) compared with controls (3.1) yielding a 1.70 hazard ratio (95% confidence interval: 1.23-2.36). Incidence rates were consistently higher for PWH in subgroups stratified by sex or the cause of the first VTE.
- PWH with lower CD4+T-cell counts at their first VTE had a lower rate of recurrent events, which was driven by PWH experiencing a better CD4+T-cell recovery on HIV treatment prior to anticoagulant discontinuation.

What do these findings mean?

- The risk of recurrent VTE is increased in PWH, but ameliorated with better immune reconstitution.
- HIV associated immunodeficiency reflects a reversible risk factor for VTE specific to PWH and is of relevance for decisions on anticoagulant therapy duration.

6. Early in the methods section of your main text, please state whether or not the study had a protocol or prespecified analysis plan, and if so attach the relevant document(s) as a supplementary file (referred to in the text). Please highlight non-prespecified analyses. Please briefly note that informed consent was obtained, around line 122.

Answer:

We have added a note on the protocol and informed consent to the first paragraph of the methods section (page 6, lines 166-168). The protocol is added to the manuscript as supplementary file. We would like to note that the initial study plan was to only include male participants. However, this was abandoned at an early stage and we chose to analyse the whole group.

The last sentence of that section now reads:

“The scientific review boards of both cohorts approved the current study. All participants gave informed consent. The study protocol is available as supplementary file.”

It is perhaps good to note that in MEGA, participants gave written informed consent, whilst in ATHENA, participants are given written and verbal information and have the option to opt out of data collection at any time. This opt-out mechanism was naturally approved by relevant ethical committees Because it was requested to briefly note that informed consent was obtained, we left the current passage as above. If the editors would like us to add this nuance to the manuscript, we would be happy to comply.

7. Please remove the "role of the funding source" statement from the text (the relevant information will appear in metadata in the event of publication).

We removed that section from the main text and put that after the main text under the heading ‘metadata’ (page 23, line 708)

8. At line 377, please adapt the text to begin "In this study, we found that ... were at higher risk ..." or similar.

We have adapted this sentence accordingly. Please refer to page 17 line 441

9. At line 396, please add "to our knowledge" or similar.

We have adapted this sentence accordingly: page 17, line 467

10. Throughout your text, please do not use italics for emphasis.

In the revised manuscript we no longer use italics for emphasis

11. At line 452 and any other relevant instances, please adapt the wording to avoid overstating the conclusions (e.g., "In conclusion, we observed a high risk of VTE recurrence in PWH.").

We have adapted the mentioned sections to the Editor’s suggestions. See page 19, line 545.

12. Throughout the paper, please format reference call-outs as follows: "... VTE recurrence [19,20].".

This has been amended throughout the manuscript.

13. We ask you to revise your reference list to ensure that citations meet journal format. For example, italics should be removed; and 6 rather than 3 author names should be listed where appropriate prior to "et al.".

This has been amended.

14. Please adapt the attached STROBE checklist so that individual items are referred to by section (e.g., "Methods") and paragraph number rather than by page or line numbers, as the latter generally change in the event of publication. Please refer to the document in the methods section.

We have updated the checklist contents accordingly. We refer to the document at the end of the methods section of the paper. (page 9, lines 335)

**Comments from the reviewers:

*** Reviewer #1:** 

I confine my remarks to statistical aspects of this paper.  These were very well done, and I recommend publication.

We would like to thank the reviewer for appreciating our study and efforts.

1. I had one question - why did the authors choose to control for variables rather than do matching or propensity scores?

This suggestion could have been a good alternative. However, we chose for the current analysis to keep it in line with earlier studies on recurrent VTE and our earlier publication (Lancet HIV 2019) which enhances comparability. Also, PWH are different from the general population and their VTE recurrence risk was our prime interest so we were not primarily interested in getting an optimally matched cohort for the analysis. Instead, we designed the study to adjust for relevant confounders by multivariable regression analysis. Furthermore, all methods have disadvantages and balancing these with their advantages made us choose for adjustment in a multivariable model.

2. Also, i commend the authors for keeping variables continuous but presenting them stratified.

Thank you

**Reviewer #2:** 

The risk of recurrent VTE in PWH is a significant question in clinical practice, especially in an unprovoked first event. This because deciding whether patients should receive indefinite anticoagulation or stop treatment as current guidelines advocate is a crucial step.
We found this this manuscript well written and organized, and capable somehow to respond to the question about risk rate of VTE recurrence in PWH further suggesting that a rapid and well reconstituted CD4+T-cell count may favor discontinuation of anticoagulation.

However, we found several unresponded questions and evidence
1) Has the role of DOACs and their interactions with modern ART therapy been assessed?

This is a relevant question. However, in this study, none of the participants were treated with a DOAC in the PWH group. In the MEGA, none were on DOAC treatment either, as DOACs were not introduced to the market at the time that the MEGA follow-up study was conducted. We added this information in the method section on page 7, lines 218-220 It reads as follows: “None of the PWH patients were treated for their VTE with direct oral anticoagulants (DOACs). None of the MEGA individuals either were treated with DOACs as DOACs were not available during the observation period.”

2) has the adherence to ART therapy during anticoagulation been assessed?

Adherence to ART has not been directly measured in our study. However, because plasma HIV-1 RNA levels are strongly correlated with adherence to ART, in clinical practice having undetectable plasma HIV-1 RNA levels can be considered an indicator of good adherence to ART.

Of the 87 people with HIV with an undetectable viral load at index VTE, 83 (95%) had an undetectable viral load at anticoagulation withdrawal. In a further 11 people, the viral load became undetectable from VTE diagnosis to anticoagulation withdrawal.

In our previous paper on risk for first VTE in people with HIV, plasma HIV-1 RNA levels were included in our regression analyses and were found to be associated with an increased risk. As explained in the methods section (page 8, lines 269), the low number of PWH without suppressed HIVRNA at index VTE prevented a meaningful analysis.

3) Patients without suppressed HIV-RNA should not be included in the study

In our opinion, including PWH with a detectable viral load still resembles a relevant clinical scenario since despite the 90-90-90 goals, still over 10 million PWH worldwide do not have sufficient access to ART and are thus viremic (<https://www.unaids.org/en/resources/fact-sheet>). As viremia is a risk factor for VTE, we can expect the proportion of viremia with people with HIV suffering a VTE to be higher than in a general cross-section of people infected with HIV. Excluding these patients as the reviewer suggests thus would preclude analyzing an unfortunately still very relevant clinical scenario.

Of course, we are only following participants enrolled in clinical practice. But here also, not all patients achieve suppressed HIV-RNA, perhaps due to problems with continuing ART treatment. As viremia induces hypercoagulability, ongoing or reversal of viremia might influence prognosis of recurrent VTE. Because our primary aim was to investigate VTE recurrence risk in the entire population of PWH who had a first VTE, we therefore chose not to exclude any particular subgroups. Many first VTE occur in patients newly diagnosed with HIV, many of them even having advanced HIV disease, and who did not yet or only recently started on ART. Indeed, 10% of the cases in our analysis suffered a VTE while having HIV but before their first visit to the HIV treatment clinic. Therefore, excluding all patient who at the time of the first VTE do not have suppressed viremia would therefore bias our results towards selecting a population doing well on ART.

Nevertheless, perhaps the reviewer is concerned that viremia is driving the conclusions regarding a higher risk of VTE recurrence compared to the control cohort. Our suspicion is the opposite: viremia at index VTE is an easily reversible risk factor, so this may actually be associated with a favorable prognosis. Unfortunately, the number of participants with very high levels of viremia, likely to have significant impact was low, and we refrained from this analysis. A sensitivity analysis excluding participants with viremia at index VTE yields the following results:

|  | **HR (95%CI) Original model*** | **HR (95%CI) only virally suppressed at index VTE** |
| --- | --- | --- |
| Overall | 1.16 (0.84-1.62) | 1.33 (0.89-1.99) |
| Follow-up < 6 years | 1.22 (0.87-1.73) | 1.32 (0.86-2.01) |
| Follow-up < 1 year | 1.67 (1.04-2.70) | 1.64 (0.89 -3.04) |
| Follow up 1-6 years | 0.94 (0.58-1.55) | 0.94 (0.53-1.68) |

We hope the results above remove these concerns. We have added these results to the supplement supplementary analyses under the heading sensitivity analyses performed after peer review.

4) We do not agree to rerun models considering only participants with CD4+T-cell below 500 at the time of index VTE, what the real impact of the dynamics of CD4+ recovery on the risk?

The underlying hypothesis that we are testing with this subgroup analysis is that VTE risk is higher in people with more advanced HIV disease and VTE recurrence rates might be lower in case clinically relevant immunodeficiency recovers substantially while on ART. This is a highly relevant hypothesis as many VTE occur in people with HIV that are newly diagnosed and not yet or only recently on ART who have moderate to severe immunodeficiency or even AIDS at the time of the VTE. However, people with HIV on ART who have CD4 counts in the normal range (i.e. above 500 cells/mm3) often exhibit large random fluctuations of their CD4 counts. These fluctuations are not reflective of immune-recovery (nor of a deterioration of their immune system in case the random fluctuation is in the opposite direction) but are instead considered of no clinical significance. By limiting this particular analysis to the subgroup of PWH who had CD4 counts below the normal range at the time of the first VTE, we increase the signal-to-noise ratio and hence our statistical power to test this hypothesis.

This has now been elaborated in the manuscript (page 9, line 324): “[…] are not reflective of immune recovery (nor of a deterioration of their immune system in case the fluctuation is in the opposite direction) and are considered of no significance in clinical practice. Therefore, models were rerun considering only participants with a CD4+ T-cell count below 500 cells/mm3 at the time of index VTE. By limiting this particular analysis to the subgroup of PWH who had CD4+ T-cell counts below 500 at the time of first VTE, increase in signal-to-noise ratio is expected.”

***** Reviewer #3:** 

Rokx et al have conducted an important study comparing the outcome of HIV patients with VTE to patients without HIV. As suggested by the authors these results have significant implications for treatment of patients with HIV and VTE. They compare outcomes in two different cohorts; the ATHENA cohort and the MEGA study cohort.

1. Although both these studies were conducted in the Netherlands, data was collected on participants at different times (ATHENA 2003-2018; MEGA 1999-2009) therefore it is possible, as noted by the investigators, that improved diagnostic methods (e.g., multidetector CT angiography, more sensitive D dimer assays) may contributed to the differences in outcomes noted. In this light, do the authors have additional information on the number of subsegmental PE in the two groups at initial diagnosis and follow up? More detailed information on the types of VTE (upper versus lower extremity DVT, lobar versus segmental vs subsegmental PE) might provide additional
evidence to support their conclusion that HIV patients have an increased risk for recurrent VTE.

We thank the reviewer for this comment. Details of the location of PE are not available in the MEGA cohort, but given that subsegmental PE was uncommonly diagnosed before 2008 (Scheres et al Res Pract Thromb Haemost2018, doi:[10.1002/rth2.12101](https://dx.doi.org/10.1002%2Frth2.12101)), while the observation period ran between 1999 and 2009, we assume that subsegmental PE, either as first or recurrent event, was rarely diagnosed in MEGA. We initially did not have information on subsegmental PE for the HIV cohort, but looked this up in chart reviews when revising the manuscript. Due to the higher risk found in ATHENA, we limited ourselves to collecting data on whether isolated subsegmental Pes had occurred as an occurrence.

Within the 40 recurrences in ATHENA, there were 21 isolated PEs. Only one was reported as subsegmental. As can be expected, exclusion of this sole event from our analysis did not materially affect our results:

|  | **HR (95%CI) Original model*** | **HR (95%CI) Excluding Subsegmental PE** |
| --- | --- | --- |
| Overall | 1.16 (0.84-1.62) | 1.13 (0.81-1.58) |
| Follow-up < 6 years | 1.22 (0.87-1.73) | 1.18 (0.84-1.68) |
| Follow-up < 1 year | 1.67 (1.04-2.70) | 1.67 (1.04 -2.70) |
| Follow up 1-6 years | 0.94 (0.58-1.55) | 0.89 (0.53-1.47) |

Additionally, a subsequent sensitivity analysis was done to explore whether any less tangible period effects would have influenced our results. This was done by choosing overlapping periods of follow-up. Ideally, we would choose overlap in index dates *and* overlap in follow-up after withdrawal of anticoagulation. However, there is only overlap between the two cohorts in index VTEs for two years (2003-2004), rendering such an approach futile as only 12% of the participants in the ATHENA cohort had their index event in these years. Therefore, we included MEGA participants who had their index event in 2003 or later (n=858) and limited follow-up time in ATHENA participants to the 31^st^ of December 2010 (n=87), excluding people with index events after this date. We reiterate that in the context of our conclusions this should be acceptable as we are mainly concerned in overascertainment of the outcome, not in overascertainment of index VTE. Also, we can reasonably assume smaller changes in diagnostic practice between 2004-2009 as opposed to a twenty year period (1999-2018). Results from the cox model are shown below. The initial time split is abandoned as data become sparse at late follow-up for both cohorts.

|  | **HR (95%CI) Original model*** | **HR (95%CI) overlapping period** |
| --- | --- | --- |
| Overall | 1.16 (0.84-1.62) | 1.32 (0.78-2.24) |
| Follow-up < 1 year | 1.67 (1.04-2.70) | 1.93 (1.04 -3.61) |

We feel that the current analysis is sufficient for its purpose, as the overall analysis does not show that hazard ratios in the original analysis have been overestimated. Indeed, hazard ratios in early follow-up appear larger, possibly due to participants in the ATHENA group have more complications as ART was started later in that era.

We hope this sufficiently addresses these concerns. We have added a section mentioning this sensitivity analysis in the methods (page 9, line 306), have listed the results appropriately (page 14, line 424) and mention this matter in the discussion (page 17, line 488). Because only the sensitivity analysis removing the subsegmental PE is more pertinent to estimating absolute risk of VTE recurrence, we have added this analysis to the manuscript. The analysis harmonizing follow-up periods has been added to the supplementary analyses appendix.

2. The authors’ finding that greater CD4 recovery was associated with a lower incidence of recurrent VTE is thought provoking. Do the authors have any information on when patients started ART? One wonders if initiation of ART at the time of VTE diagnosis is associated with a reduced risk for recurrent VTE.

We thank the reviewer for this relevant suggestion. Information on ART initiation timing is indeed available.

In our pre-specified analysis strategy, we initially only included HIV RNA and CD4 count, hereby limiting the chances of positive findings due to testing multiplicity. We chose variables that were strong risk factors for a first VTE (as identified by our analysis regarding a first VTE), represented a potentially reversible risk factor and were preferably continuous as opposed to categorical (enhanced statistical power within our small cohort with limited degrees of freedom for modelling). ART use itself was not a protective factor for first VTE (likely representing confounding by indication) and is not a continuous variable. Additionally, we thought it difficult to operationalize ART as representing some risk factor that was being reversed. The suggestion by the reviewer is therefore interesting: starting ART at a time close to the index event will likely represent participants who improve clinically between VTE diagnosis and withdrawal of anticoagulation, thereby reversing hypercoagulable factors, possibly impacting VTE recurrence risk. The limitation of this variable is however that it doesn’t quantify extent of improvement between index event and anticoagulation withdrawal.

We therefore modeled starting ART as first exposure to ART 1 month prior to index VTE or within three months of index VTE. Unfortunately, only 22 participants fulfilled this criterium, so this approach is likely underpowered. Modelling this binary variable led to the following result:

HR: 0.52, 95%CI 0.15-1.80 (adjusted for sex, age, provoked/unprovoked and infection at index VTE)

The estimated hazard ratio indeed suggests a protective effect, but the confidence interval is wide. Nevertheless, the estimate is coherent with our other results, and we thank the reviewer for this suggestion. We have added this to the supplement as a sensitivity analyses performed after peer review.

3. In the discussion I think it would be important to point out that only a small number of the PWH acquired their infections via IV drug use. I suspect that these patients may have substantially different rate of recurrent VTE than PWH acquired via sexual transmission. Studies in PWH acquired via IV drug use will be important to conduct to determine if their outcomes are similar.

We agree that a different recurrence risk in IV drug users is plausible. We have added a statement regarding this in the discussion- page 16, line 439: “ Follow-up studies should also preferentially include PWH with different characteristics regarding female sex, transmission mode (iv drug use) or from Sub-Saharan Africa regions; its affected populations’ younger age, more balanced sex ratio and higher burden of concomitant infections (e.g. malaria, tuberculosis) may further influence VTE risk.“

Minor issues
On page 16, line 321, there is a typo. I would remove "5-"
On page 18, line 353, the sentence might be clearer if written as follows "Within PWH who suffered [recurrent VTE], over 65% had a CD4+ T-cell count over 500 and 83% had an undetectable viral load at the time of their recurrence."

Response

Thank you for pointing these out. They have been adjusted in the text.

*** Reviewer #4: 

1. The authors aimed to estimate the risk of recurrent VTE in PWH compared to controls, and to identify risk factors for VTE recurrence within this population.
The most severe problem is in the study design, which could invalidate the study. The authors used two cohorts with different characteristics.

Prior to discussing what we think are in effect important potential sources of bias which may have influenced the comparative analysis between the two cohorts, it would be important to highlight several additional contextual points:

For clinical practice, the absolute risk of recurrent VTE is more important for decision making than statistical proof that the relative risk is elevated in people living with HIV compared to controls. Within the current treatment paradigm, it is therefore perhaps more important to exclude an importantly lower risk of recurrence. The lower bound of the confidence interval is instrumental to this.

In turn, most prospective cohorts on which treatment guidelines are based were carried out in the timeframe in which the MEGA follow-up study was performed. As such, it is valid to compare cumulative incidences from any future source compared to such cohorts as reference, as long as the events diagnosed are deemed clinically relevant in daily practice (which is questionable regarding isolated subsegmental PE). This should temper some concerns regarding less tangible period effects beyond subsegmental PE.

It is perhaps illustrating in this respect that cumulative incidences for participants with an unprovoked event from a large, long-term prospective follow-up study with minimal loss to follow-up (AUREC cohort, see: <https://doi.org/10.1111/jth.13524>) were quite comparable to those found in the MEGA follow-up study over both male and female subgroups. (5 year cumulative incidence males: 27% MEGA vs 30% in AUREC; females: 16.4% MEGA vs 18% in AUREC). Importantly, this study also used competing risk methods to estimate cumulative incidence- enhancing comparability. As such, the absolute risks in the MEGA follow-up study can be seen as a suitable benchmark with regards to absolute risks for recurrent VTE despite concerns inherent due to the nature of the study (follow-up and adjudication methods).

It is important to mention one caveat to this meta-argument: examination of other cohort studies reveals naturally higher and lower risks of VTE recurrence. However, even here the overall recurrent VTE rate in MEGA seems somewhat compatible to a recent meta-analysis of prospective (cohort and randomized) studies assessing recurrence risk in participants with unprovoked VTE: the 5-year cumulative incidence was 25.2% versus 24.2% in MEGA. In men, this was 28.6% in the meta-analysis and 26.9% in MEGA. However, there is heterogeneity in the meta-analysis, and this is particularly known to be an issue across studies with regards to risk difference between recurrence risk between men and women- in the AUREC study mentioned above, this risk ratio seems twofold- whilst in other studies this risk difference is negligible (Prandoni et al: [10.3324/haematol.10516](https://doi.org/10.3324/haematol.10516)). This recent meta-analysis did not investigate the reason for this heterogeneity and led to an estimate of rate ratio of 1.4 for men compared to women. Consequently, pooled cumulative incidence estimates here were somewhat higher for females compared to the MEGA study (5 year cumulative incidence: 21.2% in meta-analysis versus 16.5 in MEGA).

The point is, that the MEGA study generally appears to reflect absolute rates seen in prospective studies, especially those that see a strong difference between men and women. As such, we can assume a substantial degree of interchangeability between these data.

With this in mind, we are of course happy to address the intricacies of results within the analysis.

Consistent with the notion that the most important clinical question applies to those with unprovoked events, we now also present Cox models considering only participants with an index unprovoked event to show consistency over this clinically important stratum. (see table 3)

2. These two cohorts are from two different periods (MEGA: 1999-2009, ATHENA: 2003-2018), in which the diagnostic practices could have an influence.

We had already acknowledged the issue of possibility of differential secular trends in diagnosis and management as an issue (page 15 line 455-457). We have now additionally supplied sensitivity analyses, as outlined above (page 7, reviewer 3, item 1). We have added these results to the sensitivity analysis table. We hope these analyses address these concerns sufficiently.

3. The sample size in the two cohorts is very different. Particularly problematic is the small sample size of ATHENA cohort.

We agree with the concern about small sample. However, limited sample size mainly limits statistical power. We are not sure how this renders the study invalid: in the setting of limited power, it would mainly be questionable to conclude that no statistical ‘significance’ means no association. We mainly base our conclusions on analyses where there is moderate/strong evidence against the null hypothesis of no association. Additionally, we think that the lower bound of the confidence interval reasonably rules out an importantly lower risk of VTE recurrence.

Necessarily, the small sample size does limit the precision of the estimates found and this precluded making treatment recommendations based on immune reconstitution alone. We think that we have used language appropriate to this, as shown by

page 18 lines 527: “Finally, the number of PWH at risk became smaller with longer follow-up, consequently leading to more uncertainty of the observed estimates, precluding firmer conclusions on the risk of recurrent VTE in relation to CD4+T-cell recovery in PWH with otherwise unprovoked first VTE, as treatment advice in guidelines is driven by absolute and not relative risks. Therefore, with the current data we cannot make firm practical recommendations on the decision to continue or withhold treatment in this situation but should be seen as supportive in making a decision.”

Therefore, we also suggest that further studies should be done to facilitate a pooled analysis with our results (page 19, lines 534): “To further elucidate the influence of HIV on VTE incidence, we advise that other HIV cohorts replicate our evaluation and connect for meta-analysis.”

4. These two cohorts have different definitions of exposures.

The reviewer is correct. Therefore, we recognized this limitation throughout the article (highlighting it in the methods section, page 7, 211) and have performed sensitivity analyses specifically to address the biggest potential discrepancies in differential classification of confounding variables (table 5). With regards to assigning whether or not hospitalization only should be seen as a provoked VTE, we have modeled both extreme scenarios. Changes in hazard ratio in table 5 (1.22 vs 1.16, 1.28 vs 1.22, 1.75 vs. 1.67 and 0.99 vs 0.94 within respective time periods) do not change more than 5% and adjusted the effect of HIV upwards.

The differential definition of cancer provoked VTE is perhaps intuitively more concerning. Perhaps this necessitates some elaboration. Inherent to the definition adhered to in MEGA, it would appear that index events associated with cancer in the past 5 years would necessarily be highly sensitive but also necessarily non-specific with regards to whether cancer was actually active at the time of index VTE. Therefore, there is potential for classification of unprovoked events (conditional on no other provoking factors being present) as provoked. This is particularly important in the context of this study: there is an overrepresentation of unprovoked VTE in the HIV cohort, and underestimation of the effect of unprovoked/provoked then leads to overestimation of the effect of HIV.

We approached this by simply simulating the most extreme scenario of misclassification- reclassification to unprovoked index event was only done in the following MEGA participants:

- Those who only had cancer as a provoking factor
- Those who had a recurrence.

This led to changes of hazard ratios in the expected direction: towards null effect, but only minimally (1.16 versus 1.15, 1.22 vs 1.21, 1.67 vs 1.66, 0.94 vs 0.93 for respective time periods, see table 5). This minimal change is due to the fact that only 8 participants were redistributed.

Clearly, these analyses show that these sources of misclassification do not problematize our conclusions. The other provoking factors are factors which are routinely elicited in daily practice through questioning. Important differential misclassification between the cohorts with regards to these other provoking risk factors is highly unlikely.

5. The outcome variable (recurrent VTE) is collected differently in the two cohorts.

We used the same criteria in both cohorts to adjudicate certain events. In both cohorts, events were adjudicated primarily on the basis of clinical documentation.

In ATHENA, adjudication of certain events led to absolute cumulative estimates consistent with an advice to administer indefinite anticoagulation for unprovoked events. Four events were adjudicated as possible as detailed clinical data was missing. This was due to the notion that chart reviews are done in the HIV treatment centres, and availability of complete reports (either radiology/discharge letters) may not have been received if VTE diagnosis occurred outside the treatment centre. So, if anything, the absolute risk of VTE recurrence is likely underestimated.

In MEGA, the adjudication relied on response from patient and then ideally from centre where diagnosis was made. In the event that this was not available, a certain event was adjudicated if the recurrent VTE was clearly in a different location than the first VTE. It is difficult to appreciate whether this would lead to relative over or underestimation compared to the PWH cohort.

The sensitivity analysis considering both certain and possible recurrences is illustrative in this sense- here, the adjudication in MEGA could possibly rely on self-reports only- implying quite liberal adjudication critera and therefore very likely overestimation of VTE recurrence in MEGA relative to ATHENA. Indeed, over 21% of events were possible recurrences in MEGA whilst this was only 9% in ATHENA. Even in this fairly extreme scenario, the HR estimate for HIV infection remained higher than for MEGA.

We have reflected abovementioned nuances more clearly in the discussion. The discussion has been generally restructured to discuss both adjudication as well as loss to follow-up (as these both pertain to the same problem: ascertainment of the outcome) as a consequence to the major comment below and how this may have affected estimates. See the paragraph starting at line 471.

6. Minor comments:
The reference list should be expanded and updated (more recent publications should be included).

We have added a reference regarding the MEGA follow-up study published in 2019 in PLOS Medicine. Otherwise, we are not sure to which publications the reviewer is referring. Over 90% of the references consist from the last decade, thus seems sufficiently recent.

There are some mistakes:
- Line 75: "had lower plasma CD4+T-cell counts".

We added a space between CD4+ and T-cell

- Line 105: "supported by recent a recent study showing".

We removed the duplicate number

- Line 302: HIV specific characteristics. Align the data with the PWH column.

Response: we will defer this decision to layout editors

- Line 346: "which persisted over time (fig.2)". Figure 2 is not included in the article.
Review all the text to eliminate this type of errors.

The reviewer is right. We apologize for this omission. The figure is now included.

7. Major comments:
The percentage of women is double in the MEGA cohort. It is not a good control group. The authors should select a subpopulation that has a similar percentage of women.

We agree that that there is a disbalance of sex between the groups, however we do not think that this should be overcome by some form of matching, as

a) That will yield to a lower study sample and potential bias (random sampling assumes but does not guarantee a representative sample)
b) It is not really necessary as the regression analysis can tackle this problem by adjusting for sex.

See also to the following peer-reviewed methodology articles that concern this issue:

<https://doi.org/10.1136/bmj.i969>

<https://doi:10.1016/j.bbmt.2015.12.005>

In summary, these articles show that matching should be generally performed in studies where there are limited resources for sampling controls. The issue here is that regression analysis becomes problematic if unbalanced samples lead to small number of data within confounder strata. This leads to unnecessary inflation of estimators within a model. Matching is therefore a tool to enhance statistical efficiency: a matched sample of a given size will have higher statistical power to detect an effect than an unmatched sample of the same size. Additionally, matching can also be used as a convenient way to match on more difficult to quantify confounders. For example, by asking cases to bring spouse as control, adjustment for multiple behavioral/environmental confounders is achieved. The latter is clearly not relevant nor possible in the context of our study.

When there are sufficient data, regression is demonstrably more powerful to adjust for confounders approach because it uses all the data. Selecting a matched analysis as a default mainly discards data and, consequently, leads to loss of precision.

8. Are lost and excluded patients similar to those included in the study?

This is indeed a relevant question- to reach a judgement of whether participants with missing information different risk of recurrence had than those included.

With regards to participants who did not stop anticoagulation for various reasons we can be brief: these participants could by definition not participate in the analysis. Naturally, the fact that such continued anticoagulation, died or were lost to follow up before stopping anticoagulation informs us that these are likely to be at a higher theoretical risk of VTE recurrence than those who stopped anticoagulation. In this respect, it is illustrative to say that the relative amount of participants who could not participate due to this was higher in ATHENA compared to MEGA (24% vs 15%, respectively). Thus theoretically, more high-risk participants were ‘excluded’ from analysis in ATHENA. The theoretical selection bias would have thus led to an underestimation of effect, which is reassuring in the context of our findings. We don’t discuss this further in the manuscript because this line of reasoning is highly theoretical.

With regards to loss to follow-up, we presume the reviewer is querying the characteristics of participants who had a censoring with incomplete information and that this was potentially informative.

The number of participants censored with incomplete information were as follows:

ATHENA: loss to follow up (n=3), transferring out (n=2).

MEGA: Non-response (censored at last visit for original case-control study) (376), Documented emigration (3), Last known information at thrombosis service (participant did not respond) (32)

Therefore, in 411/4005 = 10% of MEGA participants had a potentially informative reason for censoring and 5/153= 3.2% of participants in ATHENA. Of note mortality in this context is seen as informative in the sense that these participants are no longer at risk for an event. This is accounted for in the cumulative incidence estimates.
Characteristics of these participants were as follows:

|  | **PWH complete (n= 143)** | **PWH censored* (n=5)** | **MEGA complete (n=3,594)** | **MEGA censored* (n=411)** |
| --- | --- | --- | --- | --- |
| **Male sex, n (%)** | 116 (82%) | 5 (100%) | 1,628 (45%) | 185 (45%) |
| **Age at first VTE, median years (IQR)** | 49 (42-58) | 31 (26-35) | 49 (39-58) | 48 (36-58) |
| **Location of first VTE** |  |  |  |  |
| Pulmonary embolism  (with/without DVT elsewhere), n (%) | 69 (48%) | 3 (60%) | 1473 (41%) | 164 (40%) |
| **Unprovoked first VTE, n (%)** | 100 (70%) | 3 (60%) | 1,249 (35%) | 112 (27%) |
| Undetectable viral load at index | 82 (57%) | 5 (50%) | n/a | n/a |
| CD4 median at index event (IQR) | 400  (228-600) | 178 (110-240) | n/a | n/a |
| *potentially informative censoring: transfer out, loss to follow up, starting anticoagulant treatment | | | | |

From ATHENA, emigration would appear mainly non-informative. Loss to follow-up otherwise likely also reflects interrupting treatment- which would plausibly lead to a higher risk of recurrent VTE. Thus, we can reasonably infer that there might be some underestimation of recurrence risk in the PWH cohort due to this mechanism, which is reassuring in the light of our findings. However, given the amount of loss to follow up, any effect is not likely to be very big.

In MEGA, loss to follow-up is mainly due to simple non-response and is relatively larger. It remains quite speculative how this may have biased results. It seems plausible that participants without an event would be less motivated to respond. The table supports this: we might infer that participants without a recurrence had a lower response rate due to the fact that there is a lower proportion of unprovoked index events amongst the non-responders. This would suggest an overestimation of recurrence overall in MEGA- again reassuring in the light of our findings. Even if we are mistaken, we can easily infer the implications of a worst case scenario: say the non-responders had an unplausibly 50% higher recurrence rate than the rest of the cohort. Simulating such a scenario would then lead to a 1.50*0.10= 5% relative increase of the recurrence rate of the whole MEGA cohort. Such an increase in the recurrence rate in MEGA would not change the results of the comparative analysis importantly: one can simply divide hazard ratios found by 1.05 to estimate the effect of such a simulation.

Of note, when re-examining reasons for censoring observation in ATHENA and MEGA after withdrawal of anticoagulation, we discovered that a query regarding deaths had not been updated in the database. There were 4 additional deaths in ATHENA and 5 less deaths in MEGA, this is now amended in table 1. Cumulative incidence plots were also adjusted to reflect this.

8) CD4+ T-cells at baseline were related to lower risk of VTE recurrence. How do they explain this finding? It does not seem to make sense, since low CD4+ values have been associated with a higher risk of the first VTE. In addition, immunopathological data support this association, since at lower CD4+ values, more significant inflammation and more risk of VTE.

We agree that this result seems paradoxical. The main problem with reasoning with CD4 count is that reversal of this risk factor is not binary as is the case with other transient risk factors

(i.e. surgery or estrogen use). However, the lower the CD4 count, the higher probability that some immune reconstitution will happen in the near future. Therefore, a low CD4 count at index event represents both the extent in which VTE is ‘provoked’ as well as the *potential* reversibility of this risk factor. If this risk factor is modified by the time anticoagulation is withdrawn, risk of recurrence may be low. Naturally, the CD4 count at baseline does not give information as to whether any reversal has happened when anticoagulation was withdrawn. The change in CD4 count reflect actual as opposed to potential reversal, and therefore shows a stronger association with recurrence risk.
